# Amplitude and frequency modulation of subthalamic beta oscillations jointly encode the dopaminergic state in Parkinson’s disease

**DOI:** 10.1038/s41531-022-00399-4

**Published:** 2022-10-14

**Authors:** Alberto Averna, Sara Marceglia, Alberto Priori, Guglielmo Foffani

**Affiliations:** 1 Reseach Center Aldo Ravelli for Neurotechnology and Experimental Brain Therapeutics, Dipartimento di Scienze della Salute, Università degli Studi di Milano, Milano, Italy; 2 Dipartimento di Ingegneria e Architettura, Università degli Studi di Trieste, Trieste, Italy; 3 HM CINAC (Centro Integral de Neurociencias Abarca Campal), Hospital Universitario HM Puerta del Sur, HM Hospitales, Madrid, Spain; 4 Hospital Nacional de Parapléjicos, SESCAM, Toledo, Spain; 5 Center for Networked Biomedical Research on Neurodegenerative Diseases (CIBERNED), Instituto Carlos III, Madrid, Spain; 6 Present Address: Department of Neurology, Bern University Hospital and University of Bern, Bern, Switzerland

**Keywords:** Parkinson's disease, Parkinson's disease, Neurophysiology, Neural decoding

## Abstract

Brain states in health and disease are classically defined by the power or the spontaneous amplitude modulation (AM) of neuronal oscillations in specific frequency bands. Conversely, the possible role of the spontaneous frequency modulation (FM) in defining pathophysiological brain states remains unclear. As a paradigmatic example of pathophysiological resting states, here we assessed the spontaneous AM and FM dynamics of subthalamic beta oscillations recorded in patients with Parkinson’s disease before and after levodopa administration. Even though AM and FM are mathematically independent, they displayed negatively correlated dynamics. First, AM decreased while FM increased with levodopa. Second, instantaneous amplitude and instantaneous frequency were negatively cross-correlated within dopaminergic states, with FM following AM by approximately one beta cycle. Third, AM and FM changes were also negatively correlated between dopaminergic states. Both the slow component of the FM and the fast component (i.e. the phase slips) increased after levodopa, but they differently contributed to the AM-FM correlations within and between states. Finally, AM and FM provided information about whether the patients were OFF vs. ON levodopa, with partial redundancy and with FM being more informative than AM. AM and FM of spontaneous beta oscillations can thus both separately and jointly encode the dopaminergic state in patients with Parkinson’s disease. These results suggest that resting brain states are defined not only by AM dynamics but also, and possibly more prominently, by FM dynamics of neuronal oscillations.

## Introduction

The ability to process sensory information and produce motor actions is regulated by the ongoing neurophysiological resting states of brain networks in health and disease. Large numbers of interacting neurons generate spontaneous oscillations that transition between different states, affecting neural and behavioral responses^1,2^. These oscillations are well captured by the local field potentials (LFPs) at the mesoscopic scale, which reflect the contribution of multiple current sources distributed across large populations of cells in the brain tissue^3^. Invasive LFP recordings, as well as their non-invasive electromagnetic counterparts, offer a window into the neural representation of pathophysiological states, allowing to define, predict and eventually manipulate functional and dysfunctional networks in the human brain^4–6^. Yet, the neural representation of local states at the mesoscopic scale is not completely clear.

Local brain states at rest are typically defined by the mean amplitude (or power) of LFP oscillations at given frequency bands, as assessed by standard power spectrum analysis^7–10^ and its more recent variants^11,12^. The power spectrum blends the continuous spontaneous fluctuations of both instantaneous amplitude (i.e. amplitude modulation, AM) and instantaneous frequency (i.e. frequency modulation, FM). Spontaneous AM dynamics have gained increasing attention, as they may offer mechanistic insight into the origin of LFP oscillations^13^ and can be at least as informative as the mean amplitude (or power) in differentiating brain states^14–16^. Conversely, the role of spontaneous FM dynamics in defining resting brain states remains largely unexplored. Filling this gap is important to reach a full understanding of pathophysiological states and to develop state-dependent closed-loop brain stimulation technologies^17,18^.

Here we hypothesized that spontaneous AM and FM dynamics of LFP oscillations jointly encode the pathophysiological states in the human brain. To test this hypothesis, we analyzed the LFPs recorded from deep brain stimulation (DBS) electrodes in the subthalamic nucleus (STN) of patients with Parkinson’s disease (PD) before (OFF) and after (ON) levodopa administration. The ‘OFF’ and ‘ON’ dopaminergic states in PD are a paradigmatic example of two different states with clear clinical correspondence. We specifically focused on beta oscillations (10–30 Hz), which are a biomarker of the parkinsonian OFF state and are reduced by levodopa administration with the relative clinical improvement in the ON state^19–21^. After showing that AM and FM are independent in simulated data, we examined the spontaneous AM and FM dynamics of STN beta oscillations and their dependence on the dopaminergic state in patients OFF vs ON levodopa. To gain mechanistic insight into the dopamine-dependent spontaneous FM dynamics, we then decomposed the instantaneous frequency signal into its slow and fast components (i.e. the phase slips). Finally, we used information theory to compare the relative contribution of AM and FM, as well as their synergy or redundancy in jointly encoding the dopaminergic state in PD. All results are reported, whenever possible, with Bayesian statistics (i.e. Bayes factor, BF) in parallel with standard frequentist statistics (i.e. *p*-value), because the BF provides a more equilibrated assessment of the presence or the absence of an effect^22^.

## Results

### AM and FM as independent processes in simulated oscillatory signals

**Fig. 1:**
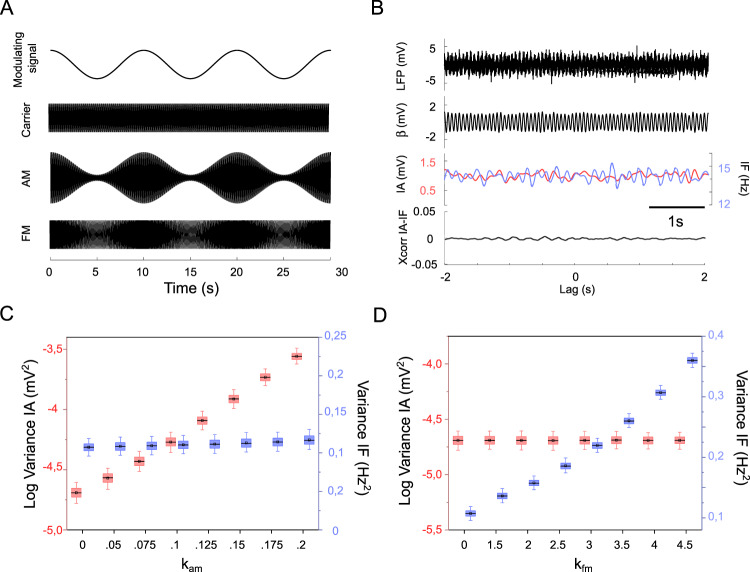
Simulated amplitude modulation (AM) and frequency modulation (FM). **A** Illustration of AM and FM. A modulating signal (0.01 Hz) is utilized to modulate the faster “carrier” sine wave (14 Hz) by means of alteration of carrier amplitude (i.e. AM) or frequency (i.e. FM). **B** Amplitude or frequency modulated sine wave combined with white noise to simulate an artificial LFP. The simulated LFP was filtered in the beta band (β, black line) and both instantaneous amplitude (IA, red line) and frequency (IF, blue line) were extracted through Hilbert transform. Bottom, cross-correlation function (Xcorr) calculated between instantaneous amplitude and instantaneous frequency. **C**, **D** Boxplots representing the distribution of the logarithmic variance of instantaneous amplitude (AM, red) and the variance of instantaneous frequency (FM, blue), as a function (**C**) of the AM index (k_AM_) or (**D**) or the FM index (k_AM_). In the boxplots, the central black line indicates the median, the central black square indicates the mean and the box limits indicate the 25th and 75th percentiles. Whiskers at ±1.5 interquartile range. As expected, changing the AM does not affect the FM and viceversa, showing that AM and FM are mathematically independent concepts.

In theoretical terms, AM and FM are two separate processes that may be independently modulated (Fig. 1A). To represent this basic concept, we simulated LFPs in which we independently modulated the instantaneous amplitude and the instantaneous frequency of a carrier oscillation in the beta range (14 Hz). We then filtered the simulated LFPs around the carrier frequency (±6.5 Hz) and applied the Hilbert transform to extract the instantaneous amplitude and instantaneous frequency (Fig. 1B). AM and FM of the simulated beta oscillations were quantified as the variance of the instantaneous amplitude and of the instantaneous frequency, respectively. In the simulations, increasing the amplitude modulation index (*k*_*am*_) produced greater AM without affecting FM (Fig. 1C). Similarly, increasing the frequency modulation index (*k*_*fm*_) produced greater FM without affecting AM (Fig. 1D). Importantly, the cross-correlation function between instantaneous amplitude and instantaneous frequency remained flat, consistently displaying no AM-FM interactions (e.g. see Fig. 1B). The absence of cross-correlation between instantaneous amplitude and instantaneous frequency was confirmed when the bandwidth of the filter around the carrier frequency was progressively decreased from ±6.5 Hz to ±3.5 Hz (Supplementary Fig. 1A). AM and FM are thus mathematically independent processes. However, this independence assumption does not necessarily hold in complex systems such as the brain, due to the possible interactions in amplitude and frequency between nearby oscillators^23,24^.

### Dopamine-dependent AM and FM of STN beta oscillations in PD

**Fig. 2:**
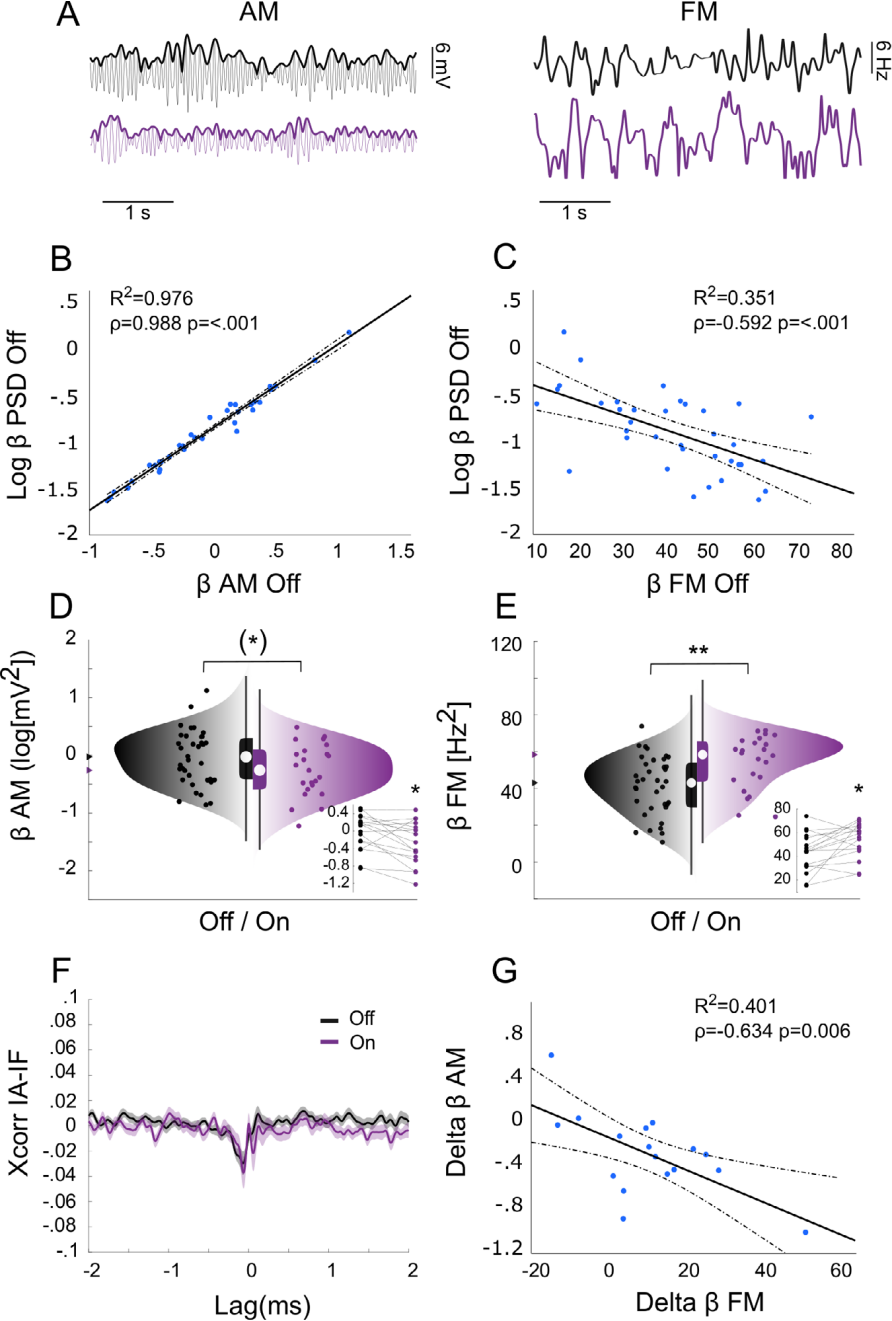
Dopamine-dependent AM and FM of STN beta oscillations in PD patients. **A** Representative signals of instantaneous amplitude (left) and instantaneous frequency (right) before (black) and after (violet) levodopa. **B**, **C** Scatter plots of logarithmic power of the beta peak (β PSD, y-axis) vs. (**B**) logarithmic variance of the instantaneous amplitude (AM, x-axis) or (**C**) variance of the instantaneous frequency (FM, *x*-axis) of the LFP filtered around the β peak across nuclei (*n* = 36) before levodopa (OFF). Thick and dotted lines are the regression lines and corresponding 95% confidence limits, respectively. **D**, **E** Raincloud plots of (**D**) AM or (**E**) FM OFF levodopa (black, 36 nuclei) and ON levodopa (violet, 23 nuclei). Insets show AM or FM changes for the subset of subjects that were recorded in both the experimental conditions (17 nuclei). Data distribution (‘cloud’), jittered raw data (‘rain’) and box plots are reported. **F** Grand-average cross-correlation function calculated between instantaneous amplitude and instantaneous frequency OFF (black) and ON (violet) levodopa. Shaded areas represent the standard error of the mean (±s.e.m). **G** Scatter plots of dopamine-dependent changes AM (delta β AM) and FM (delta β FM). Thick and dotted lines are the regression lines and corresponding 95% confidence limits, respectively. *p < 0.1 **p < 0.01. In the boxplots, the central white circle indicates the median and the box limits indicate the 25th and 75th percentiles. Whiskers at ±1.5 interquartile range. Levodopa decreased AM and increased FM, with a negative correlation between AM and FM both within and between dopaminergic states.

We then investigated the spontaneous AM and FM of STN beta oscillations recorded in PD patients at rest (Fig. 2A). We analyzed two datasets (Table 1): (i) the entire cohort (All) of 36 nuclei from 21 patients OFF medication and 23 nuclei from 14 patients ON medication (total 42 nuclei from 24 patients), and (ii) the subset of 17 nuclei from 11 patients that were recorded both OFF and ON medication (Pre-post). Grand-average and superimposed power spectra of all nuclei are provided in Supplementary Fig. 2. We again quantified AM and FM as the variance of the instantaneous amplitude and of the instantaneous frequency, respectively, of the LFPs filtered (with ± 6.5 Hz bandwidth) around the individual beta peak (14.9 ± 4.4 Hz mean ± SD, *n* = 36). All statistical results are reported in detail in Tables 2 and 3.

**Table 1:** Details of patients analyzed (24 patients, 42 nuclei).

Patient	Gender	Age (years)	Side	Recording condition	L-Dopa equivalent before surgery (mg)	Dopamine agonist dose before surgery	UPDRS III Off	UPDRS III On	β peak - Central Frequency (10–30 Hz) Off
1	F	54	R	Off, On	1500	4	66	19	11.6
2	F	69	R L	Off, On Off, On	1377	3	49	1	10.8 12.5
3	F	55	R L	Off Off	1040	2	34.5	5.5	17.2 14.0
4	M	52	R L	Off Off	2400	0	66	18.5	13.7 11.4
5	M	66	R L	Off Off	975	0.36	44.5	16	14.5 13.4
6	F	61	R L	Off Off	925	3	28	10	12.8 13.3
7	M	63	R L	Off Off	1260	1.56	37.5	4	27.6 27.3
8	M	59	R L	Off, On Off, On	1800	3	38	4	10.1 13.4
9	F	59	R L	Off Off	1671	2.34	28.5	4.4	14.0 14.5
10	F	59	R L	Off, On Off, On	1400	0	49	1	17.1 18.2
11	F	70	R L	Off, On Off, On	1200	1.8	33	2.5	12.0 10.1
12	M	66	R L	On On	900	0.7	44	7.5	- -
13	M	44	L	Off, On	1500	0	32.5	2	19.2
14	F	55	R L	On On	1250	3	37.5	4	- -
15	F	70	R L	Off, On Off, On	1010	3	36	5.5	10.5 10.1
16	M	56	L	Off, On	2800	14	39	2	16.6
17	M	38	R L	Off Off	3230	5.6	65.5	-	17.8 20.4
18	M	67	R L	Off Off	825	2.4	64	17	16.5 17.5
19	M	63	R L	On On	1292	0	11	7	- -
20	F	39	L	Off, On	800	3	72.5	2	14.9
21	F	64	R L	Off Off	1995	0	34	9	11.7 10.8
22	F	53	R	Off	900	0	68	21	10.8
23	M	4	R L	Off, On Off, On	1140	2.4	29	12	14.3 21.7
24	M	67	L	Off, On	1000	3.12	37.5	1.5	16.0

**Table 2:** Statistical results: means.

	Measure	*N*	BF	Error %	*t*	df	*p*	Cohen’s *d*
**Bayesian independent samples** ***t*-Test**	Log β AM	OFF 36 ON 23	1.0	0.006	1.8	57	0.076	0.48
	β FM	OFF 36 ON 23	13.5	0.0001	−3.1	57	0.003	−0.84
	slow β FM	OFF 36 ON 23	169.8	<0.0001	−4.1	57	<0.001	−1.09
	Phase slips β FM	OFF 36 ON 23	5.8	<0.0001	−2.8	57	0.008	−0.74
**Bayesian paired samples** ***t*-Test**	Log β AM OFF–ON	17	3.1	0.003	2.6	16	0.020	0.63
	β FM OFF–ON	17	3.7	0.003	−2.7	16	0.016	−0.66
	slow β FM OFF–ON	17	9.4	<0.0001	−3.2	16	0.005	−0.79
	Phase Slips β FM OFF–ON	17	2.2	0.005	−2.4	16	0.030	−0.58

**Table 3:** Statistical results: correlations.

	Measure	*N*	Pearson’s *r*	BF	*p*
**Bayesian Pearson’s correlations**	PSD β OFF—Log β AM OFF	36	0.99	>10^25^	<0.001
	PSD β OFF—β FM OFF	36	−0.59	218.0	<0.001
	PSD β OFF—Slow β FM OFF	36	−0.54	59.5	<0.001
	PSD β OFF—Phase Slips β FM OFF	36	−0.60	247.7	<0.001
	Delta Log β AM—Delta β FM	17	−0.63	9.3	0.006
	Delta Log β AM—Delta Slow β FM	17	−0.51	2.3	0.035
	Delta Log β AM—Delta Phase Slips β FM	17	−0.61	6.5	0.010

Across nuclei OFF medication, we observed strong positive correlation between AM and classical power of beta oscillations (*r* = 0.988, BF > 10^25^, *p* < 0.001; Fig. 2B), whereas the correlation between FM and power was negative and not as strong (*r* = −0.592, BF = 218, *p* < 0.001; Fig. 2C). Interpreting the same correlations in term of explained variance (i.e. *R*^2^), beta power explained 97.6% of the variability of beta AM, but only 35.1% of the variability of beta FM across nuclei.

AM and FM were differently modulated by the medication. We found anecdotal-to-moderate evidence for a reduction of AM after levodopa (All BF = 1.0, *p* = 0.076; Pre-Post BF = 3.1, *p* = 0.02; Fig. 2D), and a moderate-to-strong evidence for an increase of FM after levodopa (All BF = 13.5, *p* = 0.003; Pre-Post BF = 3.7, *p* = 0.016; Fig. 2E). Beta power explained 89.7% of the variability of beta AM changes, but only 30.6% of the variability of beta FM changes across dopaminergic states (data not shown).

Importantly, the cross-correlation function between instantaneous amplitude and instantaneous frequency displayed a clear negative peak both before (OFF) and after (ON) levodopa (Fig. 2F). Specifically, the FM dynamics followed the AM dynamics, on average, by about 60 ms, which corresponds to approximately one cycle of the beta oscillation. The overall levodopa-dependent changes (ON–OFF) of AM and FM also displayed moderate evidence of negative correlation (*r* = −0.634, BF = 9.3, *p* = 0.006; Fig. 2G). The negative AM-FM correlations remained stable (or even slightly decreased) as the bandwidth of the filter was decreased (Supplementary Fig. 1B, C), suggesting that they are unlikely to reflect shifts in the frequency of the beta peak in and out of the filtered band. No correlation between UPDRS III scores and beta power, AM or FM was present in this dataset, possibly because only pre-operative UPDRS scores were available (data not shown).

Spontaneous AM and FM of STN beta oscillations were thus jointly regulated, at least to some extent, both within and between dopaminergic states. AM was almost equivalent to spectral power, whereas FM seemed to partly capture a different state-dependent process.

### Slow and phase-slips components of FM

**Fig. 3:**
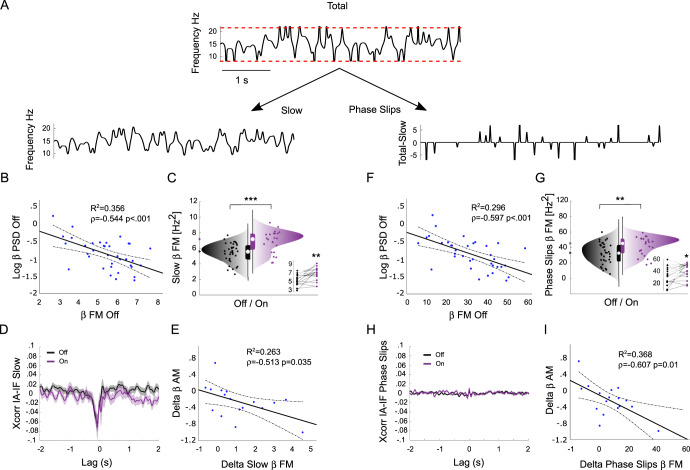
Evaluation of slow and phase-slips components of FM. **A** Representative instantaneous frequency signal before (Total) and after (Slow, left) phase slips removal, and corresponding phase-slips signal (right). Red dotted lines represent frequency limits of the band-pass filter that was used in the phase estimation procedure. **B**, **F** Scatter plots of β PSD vs. (**B**) slow FM or (**F**) phase-slips FM in nuclei recorded OFF medication. **C**, **G** Raincloud plots of (**C**) slow FM or (**G**) phase-slips FM, OFF (black) and ON (violet) levodopa. Insets show FM changes for the subset of subjects that were recorded in both the experimental conditions. **D**, **H** Cross-correlation function (Xcorr) between instantaneous amplitude (IA) and (**D**) slow or (**H**) phase-slips instantaneous frequency (IF) OFF (black) and ON (violet) levodopa. **E**, **I** Scatter plots of dopamine-dependent changes in AM (delta AM) and (**E**) slow FM or (**I**) phase-slips FM (delta FM). Thick and dotted lines are the regression lines and corresponding 95% confidence limits, respectively. **p < 0.01, ***p < 0.001. In the boxplots, the central white circle indicates the median and the box limits indicate the 25th and 75th percentiles. Whiskers at ±1.5 interquartile range. Slow FM and phase-slips FM differently contributed to the dopamine-dependent changes in FM and in the AM-FM correlations.

The instantaneous frequency can be decomposed into two main components (Fig. 3A): (i) the slower continuous fluctuations in the phase evolution of the oscillation, and (ii) the faster phase discontinuities (i.e. phase slips) (Hurtado et al.^25^). We thus investigated the specific contribution of the slow FM vs. the phase-slips FM to the overall dopamine-dependent FM and to the FM–AM correlations.

On the one hand, the slow FM reproduced most of the results we observed for the overall FM: (i) negative correlation with power (*r* = −0.544, BF = 59.5, *p* < 0.001; Fig. 3B); (ii) moderate-to-decisive evidence of increase after levodopa (All BF = 169.8, *p* < 0.001; Pre-Post BF = 9.4, *p* = 0.005; Fig. 3C); (iii) negative peak of cross-correlation between slow instantaneous frequency and instantaneous amplitude, with slow FM following AM by about 60 ms (Fig. 3D); (iv) anecdotal evidence of negative correlation between levodopa-associated changes in slow FM and AM (*r* = −0.513, BF = 2.3, *p* = 0.035; Fig. 3E). On the other hand, the phase-slips FM reproduced only in part the results observed for the overall FM: (i) negative correlation with power (*ρ* = −0.596, BF = 247.7, *p* < 0.001; Fig. 3F), (ii) anecdotal-to-moderate evidence of increase after levodopa (All BF = 5.8, *p* = 0.008; Pre-Post BF = 2.2, *p* = 0.030; Fig. 3G); (iii) no cross-correlation between phase-slips instantaneous frequency and instantaneous amplitude in correspondence to one beta cycle (Fig. 3H); (iv) moderate evidence of negative correlation between levodopa-associated changes in phase-slips FM and AM (ρ = −0.607, BF = 6.5, *p* = 0.010; Fig. 3I).

The slow and phase-slips components of FM thus differently contributed to the joint regulation of FM and AM within and between dopaminergic states.

### AM and FM information about dopaminergic states

**Fig. 4:**
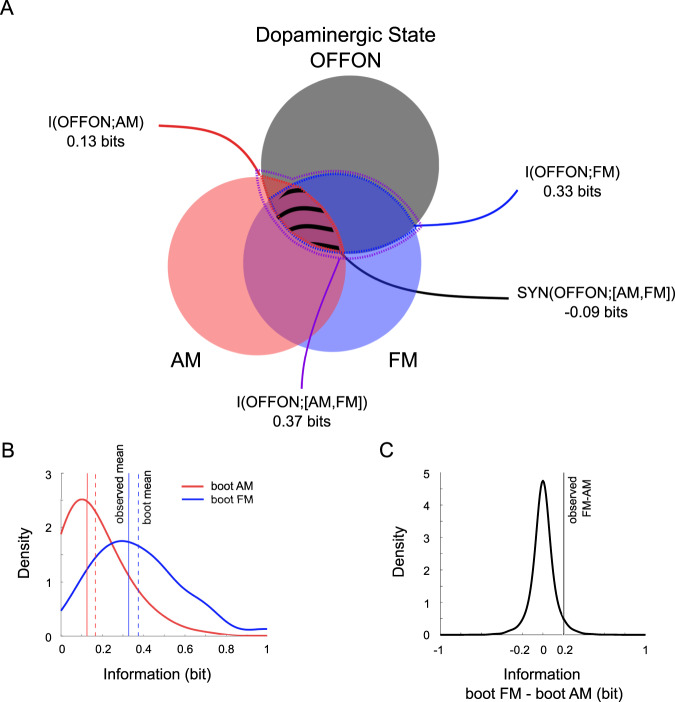
Information analysis. **A** Venn diagram representing the information carried by AM and FM about the dopaminergic state (OFFON). The black, red and blue circles represent the entropy of each variable (OFFON, AM and FM respectively). The overlap between circles represents the mutual information between variables. The areas within red, blue and violet dotted lines represent, respectively, the information carried by AM (i.e. I(ONOFF; AM)) the information carried by FM (i.e. I(ONOFF; FM)) and the information jointly carried by AM and FM (i.e. I(ONOFF; [AM,FM])). The area with black dashes represent the redundancy between AM and FM (i.e. negative SYN(ONOFF; [AM,FM])). **B** Probability density functions of the information carried by AM and by FM estimated using bootstrapping techniques. Vertical lines represent observed (solid) and bootstrapped (dashed) means for AM (red) and FM (blue) distributions. The bootstrapped means correspond well to the observed means, confirming that the bootstrapped distributions provide a meaningful representation of the variability of the estimates. The estimated distribution of FM information is greater than the estimated distribution of AM information, providing an intuitive visual representation of the difference between the two estimates (supported by formal hypothesis testing in the next panel). **C** Probability density function of differences of information between FM and AM estimated using bootstrapping techniques (i.e. under the assumption that the null hypothesis of no difference is true). The vertical line represents the observed FM-AM difference (i.e. ~0.2 bits), showing its low probability under the null hypothesis (*p* = 0.037). AM and FM convey information about dopaminergic state with partial redundancy (i.e. the information jointly conveyed by AM and FM is less than the sum of their individual contributions), with FM conveying more information than AM.

Finally, we used information theory measures to formally assess the relative contribution of AM and FM and their possible synergy/redundancy in jointly encoding the dopaminergic state in PD (Fig. 4). This approach has been extensively employed, by some of us and others, in the context of sensory processing with single-unit recordings^26–28^, and has already been applied to STN LFP recordings^29^. In the Pre-Post dataset, we specifically calculated the mutual information between the dopaminergic state (i.e. OFF or ON) and beta AM and FM, binarized with respect to the dopaminergic state (so that the marginal entropies of dopaminergic state, AM and FM are all equal to 1 bit). AM and FM information was statistically compared with bootstrapping techniques.

AM decreased with levodopa in 12 of 17 nuclei (70.6%) while FM increased in 14 of 17 nuclei (82.3%). The corresponding information jointly conveyed by AM and FM about the dopaminergic state (I(OFFON;[AM,FM]) = 0.37 bits) was moderately redundant (SYN(OFFON;[AM,FM]) = −0.09 bits; Fig. 4A). This redundancy corresponded to 27.3% of the information conveyed by FM alone (I(OFFON;FM) = 0.33 bits), but 69.2% of the information conveyed by AM alone (I(OFFON;AM) = 0.13 bits). Consequently, the information independently conveyed by FM (0.24 bits) was much higher than the information independently conveyed by AM (0.04 bits; Fig. 4A). Overall, the information conveyed by FM alone (89.2% of the total information) was significantly higher than the information conveyed by AM alone (35.1% of the total information; *p* = 0.037; Fig. 4B, C). Mutual information analysis performed in the All dataset (this time discretizing the data with 2 bits) confirmed that the information conveyed by FM (I(OFFON;FM) = 0.13 bits) was significantly higher than the information conveyed by AM (I(OFFON;AM) = 0.05 bits; *p* = 0.031). Note that the information values in the All dataset are expectedly lower compared to Pre-Post dataset due to the unpaired vs paired design.

Spontaneous AM and FM of STN beta oscillations thus jointly convey information about the dopaminergic state of PD patients at rest, with partial redundancy and with greater information provided by FM than AM.

## Discussion

We assessed the spontaneous AM and FM dynamics of STN beta oscillations at rest in PD patients OFF and ON medication. Even though AM and FM are mathematically independent concepts (Fig. 1), they displayed negatively correlated dynamics (Fig. 2): (i) AM decreased while FM increased with levodopa; (ii) instantaneous amplitude and instantaneous frequency were negatively cross-correlated within dopaminergic states, with FM following AM by approximately one beta cycle; and (iii) global AM and FM changes were also negatively correlated between dopaminergic states. Both the slow component of the FM and the fast component (i.e. the phase slips) increased after levodopa, but they differently contributed to the AM-FM correlations within and between states (Fig. 3). Finally, AM and FM provided information about the dopaminergic state of the patients (i.e. OFF vs. ON) with partial redundancy and with FM being more informative than AM (Fig. 4). The spontaneous AM and FM of neuronal oscillations can thus both separately and jointly encode the pathophysiological states in the human brain.

By analyzing the instantaneous amplitude of STN beta oscillations, we observed that AM strongly correlated with spectral power, which explained 97.6% of the variability of beta AM across nuclei and 89.7% of the variability of beta AM changes across dopaminergic states. Cortico-basal ganglia beta oscillations are abnormally increased in PD, reflecting motor impairment as well as loss of dopaminergic tone^30^. STN beta oscillations are classically quantified in the frequency domain through power spectra averaged over many oscillatory cycles^19,20,31^. More recent studies have also characterized beta oscillations in the time domain^14,32,33^, specifically as bursts of activity whose amplitude and duration contain similar information as the power spectrum about the clinical state of the patients^16,34^. Beta bursts and the AM analyzed here are closely related measures, as they both characterize the spontaneous dynamics of the instantaneous amplitude of beta oscillations: greater AM crudely corresponds to bursts with greater amplitude and longer duration. Differently from beta bursts, AM is not a threshold-dependent measure, but by definition it only captures the second-order statistics of the instantaneous amplitude. Overall, both the mean amplitude (or power) of STN beta oscillations and its spontaneous temporal dynamics describe essentially the same underlying biological process and can distinguish, at least in part, the parkinsonian (OFF) and more physiological (ON) dopaminergic states in PD.

By analyzing the instantaneous frequency of STN beta oscillations, we found that also the FM alone can distinguish the dopaminergic state of PD patients. Importantly, beta power explained only 35.1% of the variability of beta FM across nuclei and 30.6% of the variability of beta FM changes across states. The general concept of FM is highly exploited in telecommunications, due to the greater robustness of FM compared to AM against interference and signal amplitude fading phenomena. Conversely, FM is less explored compared to AM in neuroscience. In humans, apart from the encoding of frequency-modulated external signals by the auditory system^35–39^, FM of ongoing brain oscillations has been reported in at least two contexts: (i) modulation of the frequency of alpha and gamma oscillations involved in stimulus-related visual processing^40–45^; and (ii) modulation of the frequency of beta oscillations involved in movement-related processing in PD^29,46^. Using the dopaminergic states of PD patients as a paradigmatic example of pathophysiological states, the present results extend the concept of FM from the sensorimotor interaction with the external world to the coding of internal states in the resting human brain.

Spontaneous AM and FM dynamics of subthalamic beta oscillations displayed negative correlations both within and between dopaminergic states. Since AM and FM are mathematically independent measures, their correlation offers insight into the synchronization mechanisms of the underlying networks^23,24^. Of note, the slow component of the FM and the phase slips differently contributed to the AM-FM correlations.

Only the slow FM contributed to the negative AM-FM cross-correlation within dopaminergic states, with changes of instantaneous amplitude preceding changes of instantaneous frequency by approximately one beta cycle. This finding is in good agreement with observations in the rat hippocampus, where the instantaneous fluctuations in the amplitude of gamma oscillations inversely affect the frequency of the subsequent gamma cycle^47^. This negative relationship between AM and FM on a cycle-by-cyle basis mechanistically originates from the rapid adjustments in synaptic inhibition that follow the fluctuations of synaptic excitation, in order to maintain the overall excitation-inhibition balance homeostatically stable^47^. The negative AM-FM cross-correlation we observed in the STN may thus similarly reflect the adjustment of inhibitory inputs from the globus pallidus externus (GPe) through the classic indirect pathway^48–51^, balancing the fluctuations of excitatory cortico-subthalamic inputs from the hyperdirect pathway^51,52^. Interestingly, the negative AM-FM cross-correlation explained only a small fraction of the FM and was similar in the OFF and ON states, despite the overall dopaminergic state dependence of beta AM and FM. The AM-FM cross-correlation pattern of beta oscillations at rest may thus be a state-independent hallmark of the functional-anatomical organization of recurrent cortico-basal ganglia motor networks.

Phase-slips FM, on the other hand, contributed only to the AM-FM correlation between dopaminergic states, displaying no evident AM-FM cross-correlation within states. Phase slips are phase discontinuities that may reflect the synchronization readjustment of coupled oscillators^53^ and have been suggested to contribute to the initiation of beta bursts^33,54^. However, phase slips can also emerge as estimation errors when the signal-to-noise ratio of the recorded oscillation decreases^55^, which is likely to occur in the ON state after dopaminergic medication. Phase-slips FM may thus indirectly reflect, at least in part, the dopamine-dependent decrease in beta power. Overall, AM and FM seem to represent different but interacting mechanisms of neuronal synchronization that are modulated by dopaminergic state in the human STN.

Consistent with the above correlations, mutual information analysis clarified that AM and FM jointly and partly redundantly conveyed information about the dopaminergic state, and suggested that FM was actually more informative than AM in our patients. This joint AM-FM code is important both for the basic understanding of encoding and for the technological challenge of decoding the pathophysiological resting states in the human brain.

At the mesoscopic scale, AM alone does not seem sufficient to fully encode brain resting states. This is important because AM dynamics of beta oscillations correlate with BOLD signals^9^ and are thus considered as the possible electrophysiological basis of the resting-state networks^56^, which have been intensively investigated for more than two decades with resting-state functional magnetic resonance imaging (fMRI). Our results suggest that AM dynamics represent only one side of the story, because FM dynamics also contribute to encode the resting states in the human brain. Our results are thus compatible with two possible scenarios: (a) beta FM may code pathophysiological resting states by at least partly describing a different underlying biological process than beta power/AM; or (b) beta FM may describe essentially the same underlying biological process as beta power/AM, but in a more robust way (e.g. less sensitive to noise), thus possibly closer to the actual physiology. The mathematical independence between AM and FM, as well as the dopamine-independent AM-FM correlation within states, seem to favor the first scenario. In any case, FM emerges as a new robust feature to characterize pathophysiological brain states.

At the microscopic-to-mesoscopic scale, the contribution of FM highlighted here also provides some intuitive mechanistic insight. AM dynamics can be simply modeled by varying the number of neurons that are engaged at any moment in an ongoing oscillation. Conversely, FM dynamics point the attention toward the very nature of the ongoing oscillation, with more complex synchronization mechanisms requiring the interaction of weakly-coupled oscillators at nearby frequencies^57,58^. Within the context of PD, this conceptual model has been explicitly proposed for resting tremor^53,59^. Our data suggest that the interaction of weakly-coupled neuronal oscillators more generally defines the pathophysiological states in the human brain.

From a therapeutic perspective, the information provided by FM dynamics about pathophysiological brain states could be exploited for the development of state-dependent closed-loop brain stimulation technologies^17,18^. In this direction, future works should investigate whether the additional and more prominent information provided by FM over AM may extend from the binary definition of dopaminergic states to a more continuous mapping of some clinical/behavioral features. This issue seems particularly feasible to be addressed with the newer sensing devices that are becoming progressively available^60–62^. In any case, the partial redundancy of the AM-FM code seems appealing for increasing the robustness of decoding algorithms, possibly favoring the transition of closed-loop brain stimulation from controlled lab conditions to noisy real-life applications.

Overall, we propose that resting brain states are defined not only by AM dynamics but also, and possibly more prominently, by FM dynamics of neuronal oscillations at the mesoscopic scale. Our findings suggest that AM and FM of STN beta oscillations at rest jointly encode the dopaminergic state in patients with PD.

## Methods

### AM and FM in simulated oscillatory signals

We simulated amplitude-modulated and frequency-modulated signals (*s*_*AM*_ (*t*) and *s*_*FM*_ (*t*), Eqs. (1) and (2) respectively) by varying either the amplitude or the frequency of a sinusoidal carrier signal *f*_*c*_ with a modulating signal of lower frequency *f*_*m*_ (Lathi and Ding, 2010):

1 $$s_{AM}\left( t \right) = A_c\left[ {1 + \frac{{k_{am}}}{{A_m}}\cos \left( {2\pi f_mt} \right)} \right]\sin \left( {2\pi f_ct} \right) + e(t)$$

2 $$s_{FM}\left( t \right) = A_c\cos \left[ {2\pi f_ct + \frac{{k_{fm}A_m}}{{2\pi f_m}}\sin \left( {2\pi f_mt} \right)} \right] + e(t)$$

where *A*_*c*_ = 1 and *A*_*m*_ = 1 are carrier and modulating signal amplitudes while *k*_*am*_ = {0, 0.05, 0.075, 0.1,0.125, 0.15, 0.175,0.2} and *k*_*fm*_ = {0,1.5,2,2.5,3,3.5,4,4.5} represent the amplitude and frequency modulation index respectively and *e*(*t*) is a white noise. *N* = 1000 60 s long AM and FM signals were generated for each modulation index. The resulted signals were band-passed filtered ±6.5 Hz around *f*_*c*_ (14 Hz) and the Hilbert transform was used to extract the instantaneous amplitude and the instantaneous frequency. We also tested whether the results of the simulations were affected by bandwidth of the filter, from ±6.5 Hz to ±3.5 Hz.

### Ethics

The present work analyzed a dataset that was previously published^19,63^. Patients were studied after their written informed consent and approval of the ethical committee of Ospedale Maggiore Policlinico IRCCS (according to Helsinki declaration).

### Patients

As reported in Table 1, 42 nuclei from 24 patients (12 female and 12 male) with PD were studied. Patients underwent functional neurosurgery for bilateral implantation of DBS electrodes in the STN. Patients had predominantly rigid-akinetic phenotype with severe motor fluctuations. The nuclei were identified through pre-operative direct visualization using computed tomography-magnetic resonance imaging (CT-MRI) based targeting, followed by intra-operative neurophysiology with micro-recordings, intra-operative stimulation (i.e. through the exploratory electrode) and macrostimulation (i.e. through the implanted macroelectrode), and finally post-operative neuroimaging for the final assessment of the electrode position^64–68^. The implanted electrode for DBS recordings (Model 3389, Medtronic Inc., Minneapolis, USA) was composed by four metal contacts (1.27 mm in diameter, 1.5 mm in length, spaced 2 mm centre to centre), designated 0–1–2–3 in caudal-rostral direction. Electrode 0 denominates the electrode deepest in the STN and electrode 3 the one situated at the interface between the STN and the zona incerta. In all patients, the STN-DBS procedures collectively indicated that the contact pair 1–2 was within the STN^69^. DBS target stereotactic coordinates and estimated STN length were reported previously^63^.

### LFP recordings and experimental protocol

Post-operative LFPs were recorded 3 days after surgery, at rest (60–80 s) to avoid the possible influence of movements (passive or active), 12 h after withdrawal of levodopa treatment both before (off medication, 36 nuclei recorded, see Table 1) and after (on medication, 23 nuclei recorded, see Table 1) patients received dopaminergic medication (50–200 mg of oral fast-acting levodopa—Madopar Dispersibile—Roche, Monza, MI, Italy). Individual levodopa doses given during the experiment were adapted to the habitual dose of fast-acting levodopa preparation patients were taking before surgery, to ensure full clinical efficacy. On medication recordings were obtained after an experienced neurologist had determined changes in the patient’s clinical conditions, at least 30 min after medication. Differential LFP recordings were acquired simultaneously between contacts 1–2 through an analogical amplifier (Signal Conditioner Cambridge 1902, Cambridge Electronic Design, Cambridge, England). The recorded signals were amplified (x100.000) and filtered (pass-band 2–1000 Hz) then digitized (Cambridge Micro 1402, Cambridge Electronic Design), with sampling rate 2500 Hz and 12-bit quantization with 5 V range.

### LFP analysis: spectral analysis

All of the data were analyzed in Matlab R2019a (Natick, Massachusetts: The MathWorks Inc.), using the FieldTrip toolbox^70^ and custom- written scripts. Data were first visually inspected and those sections of data with gross artefacts were removed. For each patient, 60s-long epochs of LFPs were extracted at each specific experimental phase (i.e. Off and On). LFPs were normalized by subtracting the mean and dividing the result by the standard deviation of the 600–1000 Hz band-pass filtered signals. This procedure ensures matching background noise in all recordings, thus reducing signal variability^19,69^. STN oscillations at rest were quantified by LFP power spectral analysis. The power spectral density (PSD) was estimated using the Welch method with the following parameters: Windows = 5 s, overlap = 50%; DFT (Discrete Fourier Transform) points = 16,384; df (frequency resolution) = 0.15 Hz; dt (temporal resolution) = 6.55 s. We considered the beta frequency band (10–30 Hz). OFF levodopa, each nucleus was characterized to detect a peak within the whole beta range (i.e. 10–30 Hz). The frequency displaying the highest PSD value in this range was set as the central frequency of the beta peak, with an arbitrary width of ±6.5 Hz around the peak. The central frequency calculated in the recording OFF medication was maintained for analyzing the recording ON medication. For patients with only ON recordings available, the central frequency for the beta peak was set as the median frequency calculated for the entire cohort OFF medication (i.e. 14.04 Hz). The spectral power of the individualized beta band was calculated for all individual nuclei, in patients off and on medication.

The significance of spectral peaks in the beta range (i.e. 10–35 Hz) was determined as follows: (i) the linear trend (1–45 Hz) was removed from the double-logarithmic spectrogram (using Matlab function ‘detrend’)^71^; (ii) the mean and standard deviation (SD) of the PSD in the 6–45 Hz band in each nucleus were calculated; a peak was considered significant if the PSD value exceed a threshold defined by the 95% confidence interval, (mean + 1.96 SD). According to this criterion, significant beta peaks were observable in 18 of 36 nuclei OFF medication. All analyses were conservatively performed with all available nuclei, setting the central frequency of the beta peak at the highest PSD value in the 10–30 Hz range independently of its significance.

### LFP analysis: AM-FM estimation and Phase Slips detection

To extract the instantaneous amplitude and instantaneous frequency of beta oscillations, LFPs were first filtered around the individualized beta peak described above. The main results are reported with a bandwidth of ±6.5 Hz and confirmed with smaller bandwidths. A band-pass finite impulse response filter (FIR) of relatively high order (2.530) was employed to obtain good out-of-band rejection and no phase distortion. These characteristics of the filter were important for the procedure of phase slip detection (see below) because they guaranteed that the only out-of-band activity visible corresponded to singularities, thus minimizing false positives in slip detection due to poor filtering^53^. Then, given the filtered signal *z*(*t*), the Hilbert transform *H*[*z*(*t*)] was computed to extract its analytic signal (Eq. 3):

3 $$z_a\left( t \right) = z\left( t \right) + H\left[ {z\left( t \right)} \right]$$

The instantaneous phase was then calculated as reported in Eq. 4:

4 $$\phi \left( t \right) = \tan ^{ - 1}\frac{{H\left( {z\left( t \right)} \right)}}{{z\left( t \right)}}$$

and the instantaneous amplitude was computed in the complex plane as (Eq. 5):

5 $$IA\left( t \right) = \left| {z_a\left( t \right)} \right| = \sqrt {z_{Re}^2\left( t \right) + z_{Im}^2\left( t \right)}$$

Finally, Eq. 6 reports the instantaneous frequency was obtained by differentiating the time-dependent phase with respect to time:

6 $$IF\left( t \right) = \frac{{{{\Delta }}\phi \left( t \right)}}{{{{\Delta }}\left( t \right)}}$$

Amplitude modulation (AM) and frequency modulation (FM) were finally calculated in terms of deviation of the instantaneous amplitude and instantaneous frequency signals from their mean values, i.e. computing their variance (Eqs. 7 and 8 respectively):

7 $$AM = \log \left( {Var\left( {IA} \right)} \right)$$

8 $${\it{FM}} = {\it{Var}}\left( {{\it{IF}}} \right)$$

Throughout the manuscript we refer to AM as the log-transformed variance of the instantaneous amplitude, and to FM as the variance of the instantaneous frequency.

In experimental data, periodic activity is often interrupted by phase slips, resulting in discontinuities in the phase evolution of an oscillator. Phase slips appear as “spikes” in the instantaneous frequency (Fig. 3A), which exceed the frequency limits of the band-pass filter that is used in the phase construction procedure (Fig. 3A, red lines)^25,72^. The instantaneous frequency was thus divided into two main components: (i) slow FM was extracted from the instantaneous frequency by removing the phase slips (Fig. 3A) and by filling the gaps by cubic interpolation from the neighboring time points^53^; (ii) phase-slips FM was calculated by subtracting the slow FM to the raw instantaneous frequency series (Fig. 3A).

### Information carried by AM and FM

We assessed the information that AM and FM conveyed about the dopaminergic state in the nuclei recorded both OFF and ON medication (Pre-Post dataset, 17 nuclei from 11 patients), using the Information Breakdown Toolbox (ibTB)^28^ (Direct Method). By “information”, we specifically refer to Shannon’s mutual information^73,74^ between AM and/or FM and the dopaminergic state (i.e. OFF vs. ON). This is a binary problem, so the maximum information, i.e. the entropy of the dopaminergic state, is 1 bit. AM and FM were discretized within nuclei, also at 1 bit, by assigning, for each individual nucleus, a 1 to the higher value and a 0 to the lower value OFF vs ON medication. OFF and ON states were stacked so that the final dataset is composed by a by 34 × 1 array of states and either a 34 × 1 array of modulations (when AM or FM are analyzed separately) or a 34 × 2 matrix of modulations (when AM or FM are analyzed jointly). The mutual information between states *OFFON* and modulations *M* was thus calculated as follows (Eq. 9):

9 $$I\left( {OFFON;M} \right) = \mathop {\sum }\limits_s \mathop {\sum }\limits_r P\left( {OFFON} \right)P\left( {M|OFFON} \right)\log _2\left( {\frac{{P(M|OFFON)}}{{P(M)}}} \right)$$

where *P*(*OFFON*) is the prior probability of a given dopaminergic state*s* (0.5 for both OFF and ON), *P*(*M*|*OFFON*) is the conditional probability of the binarized modulation *M* (e.g. FM = 1) in a given dopaminergic state, and *P*(*M*) is the marginal probability of the binarized modulation *M* across states (also 0.5 for both 1 and 0 due to the binarization of AM and FM). *I*(*OFFON*,*M*) is expressed in bits, taking values between zero (no information) and 1 (maximum information). Note that since we were interested in comparing AM and FM information rather than in absolute information values, we did not perform bias correction for simplicity.

The mutual information analysis was extended to calculate the amount of information about dopaminergic state jointly carried by AM and FM (Eq. 10):

10 $$\begin{array}{l}I\left( {OFFON;[AM,FM]} \right) = \mathop {\sum}\limits_s {P\left( {OFFON} \right)} \mathop {\sum}\limits_{AM,FM} {P\left( {[AM,FM]|OFFON} \right)} \log _2\\\qquad\qquad\qquad\qquad\qquad\quad\;\;\left( {\frac{{P([AM,FM]|OFFON)}}{{P([AM,FM])}}} \right)\end{array}$$

where *P*([*AM*, *FM*]|OFFON) is the conditional joint probability of AM and FM (e.g. [*AM* = 0, *FM* = 1]) in a given dopaminergic state, and *P*([*AM*, *FM*]) is the marginal joint probability of AM and FM across states.

We further decomposed *I*(*OFFON*; [*AM*,*FM*]) into the sum of the information carried independently by AM and FM plus the information gained (synergy) or lost (redundancy) with their joint contribution^28,75^ (Eq. 11):

11 $$\begin{array}{l}I\left( {OFFON;[AM,FM]} \right) = I\left( {OFFON;AM} \right) + I\left( {OFFON;FM} \right)\\\qquad\qquad\qquad\qquad\qquad\quad\;\, +\, SYN\left( {OFFON;[AM,FM]} \right)\end{array}$$

where SYN indicates synergy if positive and redundancy if negative.

Finally, the mutual information separately conveyed by AM or FM about the dopaminergic states was also calculated for the entire cohort (All dataset, 36 nuclei OFF medication and 23 nuclei ON medication). In this case the data could not be discretized within nuclei (the All dataset is not paired), so we the resolution of the discretization to 2 bits (4 levels, equipopulated binning, with 0 representing the lowest values and three representing the highest values across dopaminergic states) to be able to capture the variability between nuclei, and we did not extend the analysis to the joint information and synergy/redundancy due to the limited-sampling bias associated with these analyses with the higher levels of discretization.

### Statistical analysis

Differences in AM or FM between dopaminergic states were assessed with unpaired *t*-tests in the All dataset, and with paired *t*-tests in the Pre-post dataset (Table 2). Correlations were assessed with Pearson correlation coefficient (Table 3). All statistical analyses were performed, whenever possible, with Bayesian statistics^22^ as implemented in JASP (version 0.15), using default effect size priors (Cauchy scale 0.707). Results are reported using the one-tailed Bayes factor *BF* expressing the likelihood ratio between the alternative hypothesis and the null hypothesis. Note that one-tailed testing is typically preferred in Bayesian statistics because it provides a faired balance between the ability to support the null or the alternative hypothesis^22^. Effect size estimates are reported as median posterior Cohen’s *d* using a two-tailed alternative hypothesis in order not to bias estimates in the expected direction. Evidence in favor to alternative hypothesis (*BF* > 1) or to the null hypothesis (*BF* < 1) was described according to standard levels: anecdotal (1/3 < *BF* < 3), moderate (<1/3 or >3), strong (<1/10 or >10), very strong (<1/30 or >30), decisive (<1/100 or >100). Standard *p*-values of two-tailed frequentist analyses are also reported for completeness.

For the analysis of information, we employed bootstrapping techniques for (i) estimating the probability density functions of AM and FM information to gain a sense of the variability of the estimates, and (ii) for performing hypothesis testing to formally compare the information carried by FM and by AM. Namely, to construct the probability density function we obtained 10,000 bootstrapped datasets by random resampling with replacement from the measured individual differences (On–Off) within AM and FM (taking ON and OFF values always from the same patient/nucleus), then we applied the binarization and mutual information analysis to each bootstrapped dataset. For hypothesis testing we obtained 10,000 bootstrapped pairs of datasets by random resampling with replacement from the measured individual differences (On–Off) between AM and FM (i.e. from the entire dataset of both AM and FM groups together, again taking ON and OFF values always from the same patient/nucleus, but allowing AM and FM to come from different patients/nuclei), we applied the binarization and mutual information analysis separately to each bootstrapped dataset, we estimated the *p*-value as the probability that the absolute difference between the bootstrapped datasets (bootstrap information FM—bootstrap information AM) was greater than the absolute difference between the actual FM and AM information values (information FM—information AM). For illustration purposes, probability density functions of bootstrapped AM, FM (Fig. 4B) difference of information (FM-AM, Fig. 4C) were kernel-smoothed using Matlab function ‘fitdist’ with 0.5 kernel width.

The bootstrapping analyses on the All dataset were slightly different (but conceptually equivalent). Probability density functions of information values were obtained by random resampling with replacement separately from the measured OFF or ON values within AM and FM (i.e. creating new separate vectors of resampled OFF and ON nuclei). Hypothesis testing was performed by random resampling with replacement separately from the measured OFF or ON values between AM and FM (i.e. from the entire dataset of both AM and FM groups together). Note that in this case the binning was performed before the bootstrapping.

Results are reported as mean ± SD. Box plots median (Q2), first quartile (Q1) and third quartile (Q3); the whiskers denote 1.5 times the interquartile range (IQR).

## Supplementary information

Supplementary Materials

## Data Availability

The data analyzed in the current study are available from the corresponding authors upon reasonable request.

## Code availability

Custom code used for the analyses is available from the corresponding authors upon reasonable request.
