# Supplementary material for: Amplitude and frequency modulation of subthalamic beta oscillations jointly encode the dopaminergic state in Parkinson’s disease
Source: NPJ Parkinsons Dis. 2022 Oct 14;8:131. doi: 10.1038/s41531-022-00399-4 (PMC9568523; doi:10.1038/s41531-022-00399-4)
Supplement: Supplementary file 1 — Supplementary Materials [file 41531_2022_399_MOESM1_ESM.pdf]

Supplementary Materials

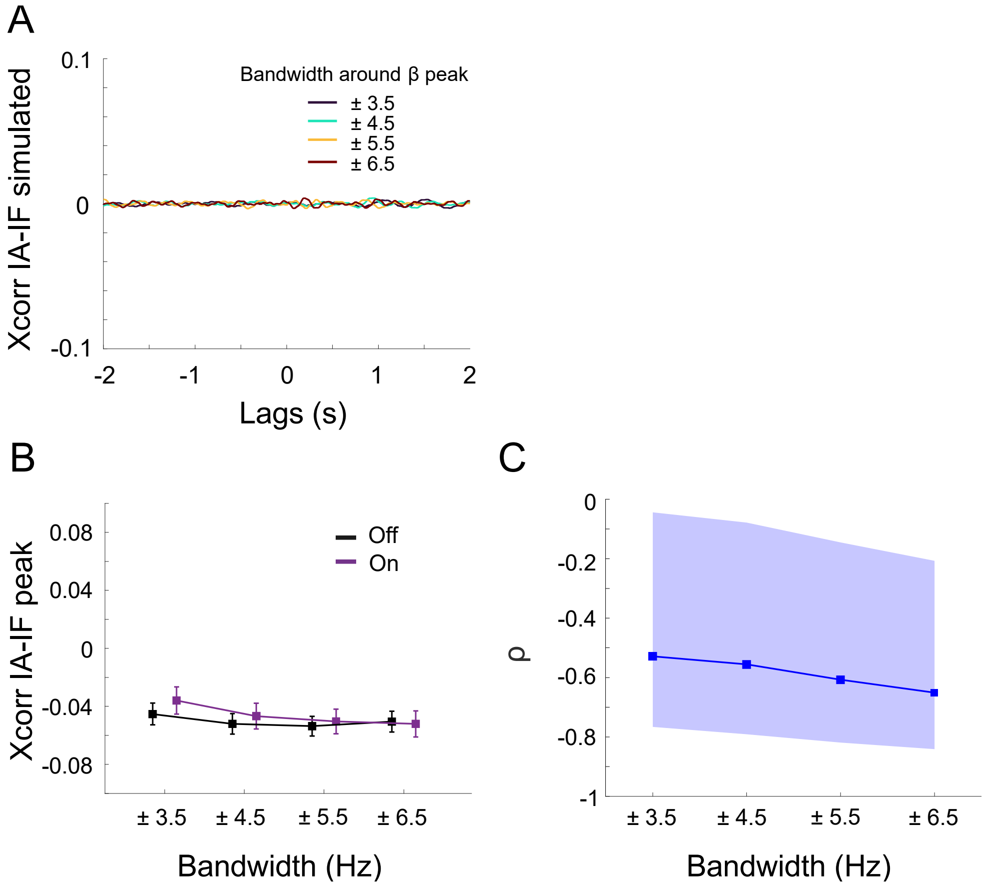

Suppplementary Figure 1: Impact of decreasing the bandwidth of the bandpass filter. A) Grand-average cross-correlation function (Xcorr) calculated between instantaneous amplitude and instantaneous frequency for simulated data (N=10000) filtered using different bandwidths (i.e. ±3.5 to ±6.5 Hz) around the beta peak B) Cross-correlation peaks (mean±SEM ±200ms around 0 lag) calculated between instantaneous amplitude and instantaneous frequency OFF (black) and ON (violet) levodopa using different bandwidths of the band-pass filter (i.e. ±3.5 to ±6.5 Hz) around the beta peak. C) Pearson correlation coefficient between dopamine- dependent changes AM (delta β AM) and FM (delta β FM) calculated using different bandwidths. The blue-shaded area displays Bayesian 95% credible intervals.

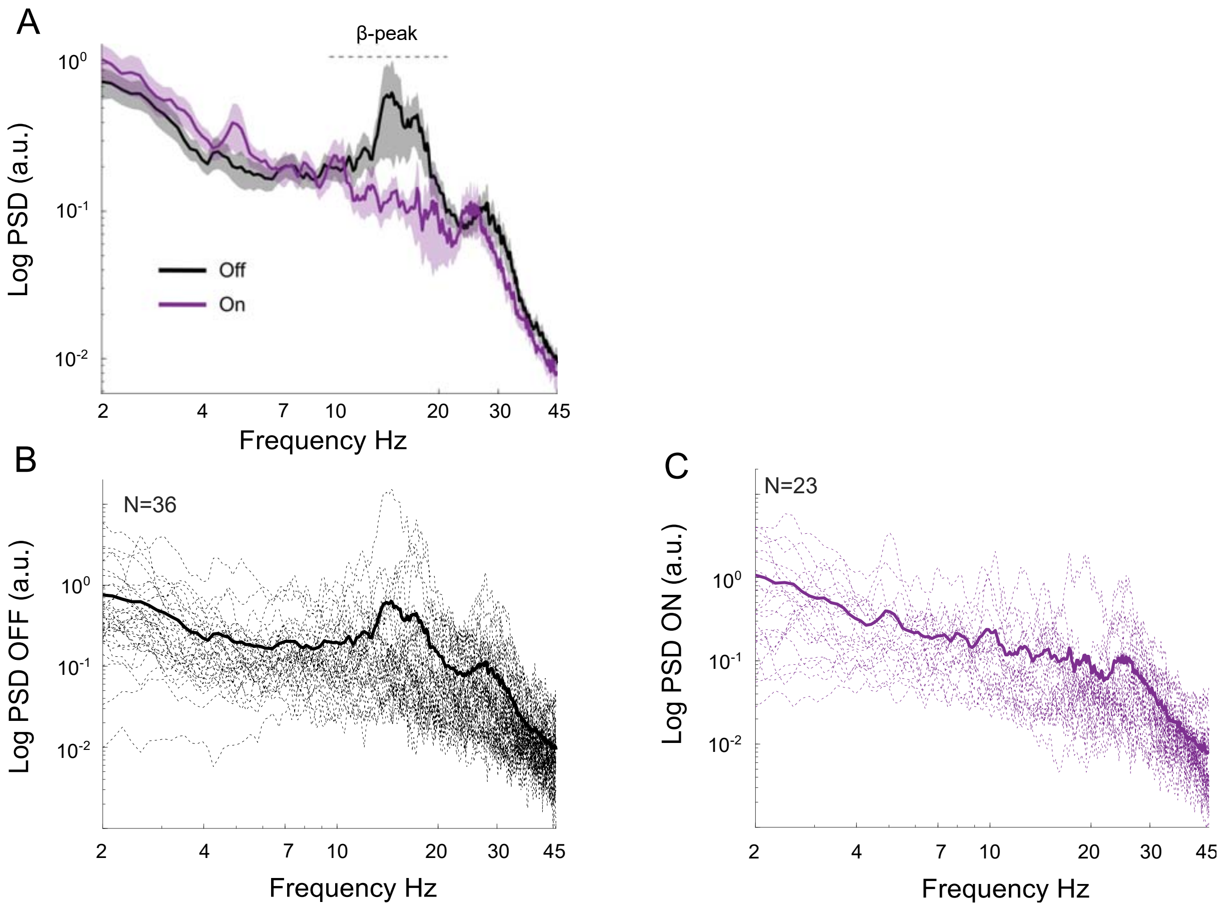

Suppplementary Figure 2: Spectral analysis of STN LFPs recorded OFF and ON levodopa. (A) Grand-average power spectral density (PSD±SEM) OFF (black) and ON (violet) levodopa in loglog scale (a.u.=arbitrary units). (B,C) Corresponding superimposed power spectral density of all nuclei (B) OFF and (C) ON medication. The bold lines represent the grand averages as in (A).
